# A pilot study: Considering spirituality in an inclusive model of practice in clinical audiology

**DOI:** 10.4102/sajcd.v65i1.552

**Published:** 2018-06-21

**Authors:** Dhanashree Pillay, Sharon Moonsamy

**Affiliations:** 1 Department of Speech and Hearing Therapy, School of Human and Community Development, University of the Witwatersrand, South Africa

## Abstract

**Background:**

A patient-orientated approach in medical clinical practice is emerging where patients and practitioners are considering and including the spiritual, emotional and psychosocial aspects of the individual. This practice is an important change in health care, specifically in the field of audiology as a holistic view of the patient now alters the perspective on the management of individuals with hearing impairments.

**Objectives:**

This article explored the experiences of a participant who reported supernatural healing of his sensorineural hearing loss (SNHL). Hence, this study focuses on the consideration of spirituality in the inclusive model of care.

**Method:**

An exploratory, qualitative narrative inquiry was used to obtain data from a single pilot case study of a 27-year-old man who reported healing of his permanent profound hearing loss.

**Results:**

Four themes were identified within the narrative obtained: prayer and faith, deaf culture, identity and purpose. The participant stated that he believed that he was partially healed to fulfil his purpose in life. The partial healing allowed him to belong to the deaf community and the hearing world simultaneously.

**Conclusion:**

South Africans live in a diverse society where most people accept spirituality as part of their search for meaning in life. Health care for individuals should therefore consider the person as a holistic being more than a medical entity. The exploration of narratives of individuals who report supernatural healing of a SNHL will assist health care practitioners and audiologists in managing individuals in an inclusive manner. This pilot study thus has implications for policy and practice in health care contexts.

## Introduction and background

Recent research is deliberating the various approaches to health care, including alternative medicine, conventional medicine, *integrative* approaches and *integral* approaches (Pan & Zhou, 2013; Ross, 2009). It can therefore be assumed that a transformation in medical clinical practice is beginning to emerge. In spite of the shift from conventional medical practice to a more holistic vision, better integration of these approaches is essential, so that best practice models are achieved for the individual (Pan & Zhou, 2013).

Different approaches to health care are discussed in the literature (Pan & Zhou, 2013; Ross, 2009). According to Ross (2009), integral medicine is the way forward from integrative medicine as it incorporates not just conventional and alternative medicine, but all dimensions of healing; from physical to psychosocial and cultural to spiritual. Pan and Zhou (2013), however, discuss integrative approaches in further depth. There are several organisations such as Hospice and HI HOPES (Home Intervention Hearing and Language Opportunities Parent Education Services) which promote holistic and integral health care, showing a better quality of life for the individual. The integral and integrative models therefore build optimal healing environments rather than focusing on a disease-centred approach and are inclusive of all systems that influence the patient. The integral model of practice spans the distance between religion, spirituality, culture, social interaction and family interaction (Ross, 2009). The basic tenet that a change in the medical clinical paradigm encourages is that even though medical practitioners may not be adept at allied health care professionals’ practices, they should nevertheless be aware of the patient’s psychological and spiritual issues and their implications when it comes to healing and health.

Limited published research into inclusive medical practices continues to keep us bound to the tried and tested gold standard of ‘double-blind randomised controlled trial’ medical practices. Furthermore, discussions on models of practice pertain mainly to medicine, with little to no research in the allied medical professions, specifically in the field of audiology.

Hearing loss – the hidden disability – is regarded as the number one malady in the world (Cohen, Labadie & Haynes, 2005); thus, a significant proportion of the world’s population is affected directly or indirectly by this communication disorder.

There are three major types of hearing loss: *conductive* (which may result from pathologies of the outer and middle ear), *sensorineural* (caused by damage to the sensitive structures of the inner ear) and *mixed* (resulting from the combination of conductive and sensorineural pathologies) (Gelfand, 2001).

Whatever the cause of a hearing loss, the most drastic effects in communication are seen in sensorineural hearing loss (SNHL). Permanent cochlear damage resulting in a SNHL may be associated with presbycusis, ototoxicity, injury, trauma, diseases and congenital aspects (Møller, 2006), with irreversible damage to the hair cells in the cochlear structure. ‘When there is irreversible loss of hearing sensitivity, it is called a permanent threshold shift (PTS)’ (Tanner, 2007). The person diagnosed with a SNHL may experience mental health issues such as depression and anxiety because ‘there is no cure’ (Eddy, 2013). Spirituality, faith and belief systems might be foundational structures of support when experiencing such mental health issues.

When considering changes in health care in relation to audiology, the impact of mental health issues should be included. The current study does not directly focus on mental health issues; however, they are included in the biopsychosocial–spiritual model adopted. There is very little international research relating to alternative methods to manage a hearing loss. The individual’s approach to health care is shaped by their religious and cultural practices. Culture plays a significant role in the way disability is viewed by an individual (Salas-Provance, Erickson & Reed, 2003) and most cultures have been influenced by science and the medical assessment and management of illnesses and disease. In the audiological context, however, there is a unique culture that has developed through the medical diagnosis of a hearing impairment. Deaf culture is a collective group of deaf individuals who identify themselves as having a common language and an understanding of each other (Siple, 2003). The notion of ‘hearing loss’ is changing as holistic management aims to be inclusive of all areas that are central to the patient. The question that has sparked this change is: *What can be lost that was never there in the first place?*

The ‘representation of the self’ (Baumeister & Vohs, 2003) is the most common way of developing an identity. Individuals group themselves based on morals, spiritual values, religion, profession, tribes and family names amongst others. Deaf culture is nevertheless the only *medically defined culture* as it is based solely on the premise of a hearing impairment. Parents are the biggest role-players when determining if a child will develop a specific form of communication modality. Even though signing is the major method used by individuals who belong to the deaf culture (Ladd, 2003), most parents opt for some method of medical management to improve the hearing ability of their child.

International research focuses on hearing aids and implant technology as a priority for the audiologist and an aim of successful audiological intervention. Rehabilitation, according to medical sciences, is said to be the only option to improve hearing and communication for individuals with a permanent SNHL. According to the authors of this article, however, this view seems narrow in the context of rapidly changing perspectives in world health care.

As there is little published research in audiology, the significance of the current study will contribute to the literature, not just in a South African context but to medical practice globally. Integrative models of practice are complex and require a mental shift from a straightforward administration of a medical perspective in audiology to a mindfulness of the person and the systems in which that person operates. The theories of person and environment, as well as biopsychosocial models are recommended rather than merely a medical model perspective. Despite the changes and influences from the world, each individual has personal standards, morals and spiritual values and these serve as internal regulators for reshaping ideas and actions based on the circumstance (Bandura, 1991). Social theoretical models have agents that intentionally influence the individual’s life. These agents could be a *direct personal agency, proxy agency* or a *group agency* (Bandura, 2002).

Spiritual and religious leaders, therapists, doctors and family members act as proxies and they play a major role in the well-being of the individual concerned. The actions of others in contributing to decisions taken are important as these actions or decisions form supportive bases in the spheres of healing and rehabilitation. Through the social learning system, new patterns of behaviour are acquired by direct experiences of healing or through observation of healing in other individuals (Bandura, 1977). By understanding the human psychosocial relationship, the audiologist can aim to provide a more inclusive service delivery to all patients regardless of race, religion, spiritual beliefs and background. The key to the inclusive approach selected is that the patient is involved and is an active part of the decision-making process, and thus active in their healing. This article argues that the conventional medical model used in medical practices, specifically in audiology, should change to embrace a more inclusive model that is patient-centred, encompassing mind, body and spirit. This inclusive model of practice in audiology should focus on the potential for mental, emotional, social, behavioural and spiritual processes that affect a patient’s health, personal growth and quality of life.

This article thus examines an inclusive model of intervention to patients with a hearing loss who report on spiritual healing, as documented narratives of patients are lacking in the literature. The diversity of cultures and spiritual beliefs within South Africa encourages the need for such research so that the experiences of these individuals can be documented. The diverse South African population seeks advice from traditional healers, pastors, rabbis, hakeems and gurus as a source of healing and these alternative healers should form part of the holistic management programme.

Audiology should not merely be defined by the practice of assessment and management of a hearing loss. Hearing loss affects a person’s life, including (where they exist) their religious beliefs and practices, and a person’s faith. These contribute to the biggest influences in that individual’s reactions to life events, including illness and disease. Spirituality is one of the key strengths in personal well-being (Ellison & Levin, 1998) and should be taken into account in the audiological intervention process.

## Research method and design

### Aims of the study

The main aim of this study was to explore and document the narratives of South African individuals who reported a supernatural healing of a SNHL.

The following sub-aims operationalise the main aim:

- documenting the ‘turning point’ event or events that caused the reported healing to occur
- documenting the participant’s perceptions of the mechanism of the healing
- describing the participant’s perceptions of the reactions of other people to the reported healing
- documenting the social influences of spirituality and beliefs on the healing process in audiology.

### Sample

The sample for the main study consists of between 8 and 10 participants, using purposive and snowball sampling. Narrative researchers can select a small sample size to achieve adequate information from in-depth interviews that answers the research questions (Jones, Brown & Holloway, 2012). The researchers aimed to balance the distribution of participants from the major religious and racial groups in South Africa. Participants who had been diagnosed by an audiologist with any degree of SNHL, either unilaterally or bilaterally, were included in the study. This article reports on the pilot study of this main study. A 27-year-old man narrated his story.

### Design and instrumentation

An exploratory, qualitative research design was used. This study was exploratory in nature as supernatural intervention has had limited theoretical analysis and is an area of undocumented occurrences (Sim & Wright, 2000) in South African medical practice, particularly within the context of audiology. A narrative inquiry interview was used to obtain data. The narrative inquiry included records, stories and ideas that provide information about the experiences of the individual (Mitchell & Egudo, 2003). A sound understanding of human behaviour and the rationale for such behaviour becomes evident in a qualitative study (Wisker, 2007) and the current study aimed to document individuals’ experiences of supernatural healing shaped by a belief system, a behaviour pattern and by society as a whole. An interpretive approach was adopted as the participants’ experiences are being studied within their respective contexts taking into consideration the subjective meaning to the context (Willis, 2008).

### Procedure

A semistructured interview was used (Connelly & Clandinin, 1990) to obtain narrative data from individuals who reported having experienced supernatural healing. The interview began with an open-ended question that allowed the participant to narrate his or her experiences of the healing process. Additional questions were asked when necessary. The interview session was recorded to ensure that the information was captured accurately, thereby increasing the credibility and trustworthiness of the data. The rapport between the researcher and the participant was maintained throughout the interview process (Immy, 2005).

### Analysis

The data were transcribed by the researcher and thematic content analysis was conducted on the transcribed information. Thematic content analysis is a significant area of analysis within narrative analysis, where common themes of the individuals’ experiences and perceptions are extracted regarding (in this instance) the supernatural intervention and healing process (Symon & Cassell, 2012). The narratives were initially deconstructed from qualitative raw data transcriptions into words or sentences that were coded. According to Kim (2015), the codes were grouped into familiar categories and within each category, themes were identified.

This study met all the required ethical parameters as set by the University of the Witwatersrand’s Human Ethics Committee (protocol number H15/02/07).

## Results

A single pilot case study was conducted with a 27-year-old South African man, who reported experiencing supernatural healing of the hearing in one ear at the age of 7. The participant was diagnosed with a profound bilateral SNHL at birth and attended a school for deaf children. The participant stated that he was diagnosed and fitted with bilateral hearing aids; however, he did not use the one hearing aid post-healing. The focus of this study was to document the participant’s experiences of the ‘healing’ in relation to the hearing loss. The study did not aim to prove that the hearing loss was ‘cured’; therefore, a new audiogram was not required as proof of the recovery of hearing.

The participant is of the Christian faith and he reported that the hearing in his left ear was healed, and his healing, he believed, was an answer to the prayers of his family:

‘I was six years old or seven years old … I started to hear on the right, no on the left ear (pointing to the left ear). So far I’m happy, I’m Christian, and I know God always helps me to hear. Maybe later on He (God) will heal me 100%, because now I’m growing up now I know sign language and I’m English speaking. And I believe God helped me to speak and to hear. Even though I cannot hear in right ear, nothing in the right ear, only the left ear. I can speak, show the people how I talk, how I prove how I feel God’s presence.’ [P1, Male, 27 years]

‘So I understand different situations, I’m happy with God, I always pray. My family they (are) Christians, my uncle and my cousin are pastors. I have another uncle that is a pastor too. There are so many people who are Christian in my family. And I realised that they were praying for me, God answered their prayers to improve my life and I received lots…’ [P1, Male, 27 years]

From this narrative, the importance of identity with the deaf is evident. This participant holds a strong deaf identity, as he relates, communicates and identifies with the deaf culture; however, he also continues to see himself as a hard-of-hearing individual. He stated that he feels:

‘God helped me to hear partially because there’s so many Deaf people who don’t know God and so God was giving me purpose, to communicate with Deaf people and hearing people, that’s what I feel. So I share the Word of God, about God with the people whose hearing and Deaf. So far I’m happy that I still stayed hard-of-hearing because I can communicate with this side and this side (meaning with the hearing and the hearing impaired). If, imagine if God healed me 100% full I wouldn’t have talked with Deaf people about God, cos they don’t know. I feel God’s purpose for me to be hard-of-hearing and I’m happy with who I am. So I’m happy that I’m hard-of-hearing and I can hear a little bit and I know Deaf Culture, Deaf feelings, how the people feel if they don’t hear and how the people who hear, how they feel. So I understand different situations, I’m happy with God, I always pray.’ [P1, Male, 27 years]

This participant’s identity is driven by his ability to define this healing event as a purpose-driven event in his life.

Human interaction and relationship-building dictate that it is inevitable that there will be questions surrounding such claims of healing. This participant had a friend of a different faith, who questioned his healing and the mechanisms behind it as the friend was curious about the reasons for the healing.

‘My one friend is Muslim, he don’t believe in God, how I believe, how I, he asked where my hearing comes from. Maybe spirit, coming from God? People they can’t see God, the spirit, the relationship, the feeling, even hearing from God is a must. But must get the information in the heart, in my mind when you read the Bible, when you ask something to God and you are rushing to get the answer, one day when I was reading the Bible, that’s the answer (pointing to the page in a book). Then I see the Bible is the truth and now many people don’t believe the Bible, (they ask) who wrote the Bible, how he gets the words from? And I try to explain, people who wrote because they get their message from God, through hearing, dreams…’ [P1, Male, 27 years]

From the results of the participant in the pilot study, several themes have emerged that confirm or support findings discussed in the literature. The following four themes are discussed below: prayer and faith, deaf culture, identity and purpose.

## Discussion

### Prayer and faith

Christians believe in God – The Father, The Son and the Holy Spirit. When individuals proclaim Jesus as their Lord and Saviour, then the believer is ‘filled’ with the Holy Spirit of God (Ware, 2005). Christianity reveals that the Holy Spirit is the agent for healing and testimonies are used to support the belief that the healing is a Godly intervention at work (Broadman & Holman, 1998). Faith healings are believed to occur through prayer and the *laying on of hands* (Christensen & Kockrow, 2013). Christians believe that disease and illness are not from God and believers practice the physical *laying on of their hands* on the sick person, while praying for the power of God to move through them and heal the individual (Porterfield, 2005).

This participant stated that he believes that God healed him as an answer to the prayers of his family members. This indicates that an action was required to elicit a response from God, and the active spiritual belief, faith and prayers of others led to the supernatural healing. The narrative in this study can motivate the audiologist to self-reflect on the following questions:

Should audiologists be conversant with the spiritual beliefs of their patients? Should audiologists include questions in the case history session that would direct the patient into divulging information pertaining to their spiritual beliefs and non-medical practices?

The information obtained by the audiologist can be shared with the ear, nose and throat (ENT) specialist within the multidisciplinary team. The ENT can use the information obtained by the audiologist to benefit his or her own assessment and management practices. If we believe that the medical clinical model is failing the patient (as the medical clinical model appears to focus more on the disease than the person), then we need a shift in mind-set. This shift means that audiologists need to be moving towards a goal of including questions relating to faith and spiritual beliefs from the initial consultation with the patient.

### Deaf culture, identity and purpose

Deaf culture, identity and purpose are themes that conjure up further questions for us as audiologists. Are we defined by the illness or disease? Who are we and why are we experiencing these situations in life? Are we designed to teach others about perseverance as part of the process of overcoming difficult situations?

The purpose of life is an ongoing enigma for every individual. People tend to rationalise good and bad events so that it allows them the ability to accept the situation at hand and move on. The 27-year-old participant in this study referred to his deafness as a positive factor in his life as he stated that he believes that he can communicate with the deaf, and impact them by sharing information about God. His purpose was defined as the provider of life-changing information to the deaf. The participant stated that he was bilingual as he is proficient in both sign and oral language. Furthermore, he sees himself as a bridge between the deaf and hearing worlds. He reiterates that there is a reason for his partial hearing as he now belongs to a ‘bicultural’ world, straddling the hearing and deaf communities allowing him to share his knowledge about God. Cultural and linguistic factors are therefore important considerations in medical clinical management.

Similar to the participant in the current study, research has indicated that long-term breast cancer survivors reported a deeper spiritual experience following their diagnosis (Sabado & Tanjasiri, 2010). The findings from the current pilot study indicated that the person as a whole matters more than simply his or her disease or disability.

## Conclusion

Change is required from a medical clinical model to a more inclusive model of intervention within health care and this has been recommended in the literature. Increased reports of more ‘personal experience evidence’, is, however, now available to support transformation in intervention approaches. This study therefore argues for an inclusive approach to intervention in audiology.

We live in a spiritual society and most people accept a spiritual aspect in their lives as they seek to obtain a meaning of life (Bandura, 2003). The medical model dictates the service delivery within the health care sector, excluding the psychosocial and spiritual aspects of the patient’s life. When health care provision negates the holistic and phenomenological perspectives, it is antithetical in its approach to understanding the patient as a whole. Therefore, health care for individuals should be inclusive of the whole person and to not only be seen as a medical entity. The history of patient care indicates that the majority of patients are assessed and managed under a medical model which focuses on the doctor or therapist’s education and knowledge when assisting the patient. An inclusive perspective of audiological management should therefore shift the focus from the disease to the person.

The medical model has led to the person being seen as a specimen and despite the advances in cracking the genetic code, the understanding of the human as a whole has stagnated (Sulmasy, 2002). As far back as 1988, researchers such as McLeroy, Bibeau, Steckler and Glanz began to discuss the notion of an ecological model of patient care. A holistic model that focuses on the community, public policy, social and individual influencers is required. How should audiologists then respond to the patient in a holistic manner? The audiologist can work holistically with a patient by obtaining the support of a multidisciplinary team, consisting of counsellors, psychologists, religious leaders or community leaders. Psychosocial–spiritual factors of influence will dictate the compliance of patients to hearing screening, hearing aid assessments and management. The impact of a hearing loss stretches far beyond the borders of the home environment. Management and care of the patient should thus also traverse beyond the borders of the hospital.

Sulmasy (2002) supports the holistic biopsychosocial–spiritual model of care, as patients stated that they would like their spiritual needs to be considered when they are being managed by a health professional. A holistic model of patient care should include significant role-players in the patient’s spiritual community. McLeroy, Bibeau, Steckler and Glanz (1988) define ‘community’ in three ways, namely as a mediating structure to which the individual belongs, the relationships amongst groups in a defined area and as a geographical term.

The mediating structure of community is vital as the community is a source of social resource and social identity (McLeroy et al., 1988) that is required by the hard-of-hearing or deaf individual. Griffin (2013) stated that integrated models of support and care will shape the patient’s future as well as the future of country as a whole.

The following questions are relevant and should be reflected on to create the shift in health care practice:

- How do we transition from the old to the new inclusive health care process?
- Are audiologists equipped and ready to change the way we assess and manage our patients?

Audiologists are making progress and the evidence must be documented in further studies within the areas of supernatural healing and medicine. This study should therefore contribute further to understanding the changes needed in medical clinical practice.
